# Transition of a Text-Based Insulin Titration Program From a Randomized Controlled Trial Into Real-World Settings: Implementation Study

**DOI:** 10.2196/jmir.9515

**Published:** 2018-03-19

**Authors:** Natalie Koch Levy, Natasha A Orzeck-Byrnes, Sneha R Aidasani, Dana N Moloney, Lisa H Nguyen, Agnes Park, Lu Hu, Aisha T Langford, Binhuan Wang, Mary Ann Sevick, Erin S Rogers

**Affiliations:** ^1^ Division of General Internal Medicine and Clinical Innovation Department of Medicine New York University School of Medicine New York, NY United States; ^2^ Department of Population Health New York University School of Medicine New York, NY United States

**Keywords:** insulin/long-acting/administration & dosage, diabetes mellitus, type 2/drug therapy, medically underserved area, telemedicine, healthcare disparities

## Abstract

**Background:**

The Mobile Insulin Titration Intervention (MITI) program helps patients with type 2 diabetes find their correct basal insulin dose without in-person care. Requiring only basic cell phone technology (text messages and phone calls), MITI is highly accessible to patients receiving care in safety-net settings. MITI was shown in a randomized controlled trial (RCT) to be efficacious at a New York City (NYC) safety-net clinic where patients often have challenges coming for in-person care. In 2016, MITI was implemented as usual care at Bellevue Hospital (the site of the original RCT) and at Gouverneur Health (a second NYC safety-net clinic) under 2 different staffing models.

**Objective:**

This implementation study examined MITI’s transition into real-world settings. To understand MITI’s flexibility, generalizability, and acceptability among patients and providers, we evaluated whether MITI continued to produce positive outcomes in expanded underserved populations, outside of an RCT setting.

**Methods:**

Patients enrolled in MITI received weekday text messages asking for their fasting blood glucose (FBG) values and a weekly titration call. The goal was for patients to reach their optimal insulin dose (OID), defined either as the dose of once-daily basal insulin required to achieve either an FBG of 80-130 mg/dL (4.4-7.2 mmol/L) or as the reaching of the maximum dose of 50 units. After 12 weeks, if OID was not reached, the patients were asked to return to the clinic for in-person care and titration. MITI program outcomes, clinical outcomes, process outcomes, and patient satisfaction were assessed.

**Results:**

MITI was successful at both sites, each with a different staffing model. Providers referred 170 patients to the program—129 of whom (75.9%, 129/170) were eligible. Of these, 113 (87.6%, 113/129) enrolled. Moreover, 84.1% (95/113) of patients reached their OID, and they did so in an average of 24 days. Clinical outcomes show that mean FBG levels fell from 209 mg/dL (11.6 mmol/L) to 141 mg/dL (7.8 mmol/L), *P<*.001. HbA_1c_ levels fell from 11.4% (101 mmol/mol) to 10.0% (86 mmol/mol), *P*<.001. Process outcomes show that 90.1% of MITI’s text message prompts received a response, nurses connected with patients 81.9% of weeks to provide titration instructions, and 85% of attending physicians made at least one referral to the MITI program. Satisfaction surveys showed that most patients felt comfortable sharing information over text and felt the texts reminded them to take their insulin, check their sugar, and make healthy food choices.

**Conclusions:**

This implementation study showed MITI to have continued success after transitioning from an RCT program into real-world settings. MITI showed itself to be flexible and generalizable as it easily fits into a second site staffed by general medical clinic–registered nurses and remained acceptable to patients and staff who had high levels of engagement with the program.

## Introduction

### Background

According to the Centers for Disease Control and Prevention, as of 2015, 30.3 million people in the United States (9.4% of the population) had diabetes [1]. By 2050, the prevalence of the disease is expected to rise, affecting as many as 1 in 3 Americans [2]. Moreover, diabetes is one of the costliest diseases in the United States [3]. In 2012, the total cost associated with the disease was US $245 billion, including US $176 billion in direct medical care costs [4]. Diabetes puts patients at risk for blindness, kidney failure, nontraumatic limb amputation, stroke, and coronary heart disease [5-10]. Controlling blood sugar can help prevent the long-term adverse health consequences of diabetes [7-11].

Prior research has found significant disparities in the burden of diabetes, such that the prevalence is much higher in racial and ethnic minorities than whites [1]. Compounding the disparity in disease burden, racial and ethnic minorities have less access to care than whites [12]. Taken together, racial and ethnic minorities suffer disproportionately from the negative health consequences of uncontrolled diabetes [1,12,13].

The majority of patients with diabetes can control their blood sugar with lifestyle modifications and oral medications. However, more than 25% of patients in the United States with diabetes take insulin [14]. When insulin is needed, the first type of insulin started is basal insulin, which is in charge of controlling the blood sugar in the fasting state, for example, while we sleep. Patients with type 2 diabetes starting on a given dose of basal insulin are asked to check their morning fasting blood sugar at home daily while taking that particular dose [15]. Then, the dose of basal insulin is gradually increased (or *titrated*) until the morning fasting blood sugar falls into the desired range [15]. Ideally, a patient’s dose of basal insulin will bring all of the fasting sugars into the desired range. In reality, patients have variability in factors such as food intake and energy expenditure from day to day, which creates variable fasting blood sugars. It is important to note that basal insulin titration is limited by the lowest sugar. Thus, once any morning fasting blood sugar falls into the desired range, caution must be exercised with any further increase in basal insulin to avoid hypoglycemia.

Of note, in this scenario variability, more insulin may be needed on many days, but it will need to come in the form of a second type of insulin referred to as mealtime insulin (or “bolus”) insulin. Unlike the basal insulin discussed above that is given at a consistent dose each night, mealtime insulin is rapid acting and of short duration. Bolus insulin doses, therefore, are adjusted in real time from meal to meal depending on the blood sugar of the moment. Patients with diabetes needing titration of basal insulin have the option of self-titration [16]. Patients who are not comfortable with self-titration usually return for in-person interactions with their provider. In-person titration requires that frequent appointments be available and that patients are able to attend such appointments.

Safety-net clinics serve a predominantly ethnic minority population who face disproportionate logistical challenges to in-person care (ie, missed-work leading to lost wages, transportation challenges) [12,13,17]. Within this population, remote adjustment of basal insulin for patients with diabetes is an appealing option.

### Prior Work

Many cell phone–based programs do exist to help patients track their blood glucose, but we found no programs that only required *basic* cell phone technology to *both* gather blood glucose information and give titration advice [18-25]. To address this, the Mobile Insulin Titration Intervention (MITI) was created [26]. The goal of MITI was to provide a remote basal insulin titration program for patients with type 2 diabetes initiating or titrating basal insulin, utilizing the basic, low-cost, cell phone technology that most patients in safety-net clinics are already using [18-25]: text messages and phone calls.

Patients enrolled in MITI still have to inject insulin and monitor their fasting blood glucose (FBG). Clinicians still need to advise patients on how to adjust their once-daily basal insulin dose. However, with MITI, instead of patients having to travel to the clinic to exchange this information in-person, the clinic comes to the patients through their cell phones.

MITI patients receive a daily weekday text message asking for them to type back their fasting blood sugar and a weekly phone call from a registered nurse advising them on how to adjust their basal insulin dose. Upward titration of basal insulin through the MITI program stops when *one* morning fasting blood sugar level falls into the desired range of 80-130 mg/dL (4.4-7.2 mmol/L), when the patient reaches a basal insulin dose of 50 units, when a patient withdraws from the program, or when 12 weeks elapse. MITI is not designed to be an indefinite program. Rather, it is a (maximum) 12-week opportunity to have one’s once-daily basal insulin adjusted in a patient-centered, convenient way. If at the end of 12 weeks, the blood glucose is not controlled, the patient needs to return for in-person evaluation and titration.

MITI was successfully pilot tested in a randomized controlled trial (RCT) with 61 patients and shown to be efficacious at Bellevue Hospital (the oldest public hospital in the United States) in 2013 and 2014 [26]. In total, 88% percent of pilot study patients (compared with 37% of control patients) were able to find their optimal insulin dose (OID), and they did so in an average of 3 weeks. Additionally, 84% of text message prompts received a response and patients had high satisfaction with the program. On the basis of the success of the pilot study, from April 2016 to April 2017, MITI was implemented into usual care at both Bellevue Hospital and another New York City (NYC) Health and Hospitals site, Gouverneur Health.

A systematic review and network meta-analysis of the comparative effectiveness of different telemedicine strategies was published in October 2017 by Lee et al [27]. The group evaluated 107 telemedicine studies involving over 20,000 patients. Studies focusing on tele-education, telemonitoring, and teleconsultation led to improved HbA_1c_, with the latter (teleconsultation) being the most effective. Their conclusion was that “assessing the acceptability and implementation challenges of telemedicine in resource-poor areas is an important next step to accelerate translation.” This study directly addresses this need for more implementation evaluations in the field of telemedicine.

### Goal of This Study

It can take 17 years for clinical interventions to transition from efficacious RCTs to routine clinical practice, and many programs shown to be efficacious during RCTs never make it into usual care [28]. Implementation science studies ways to promote the adoption of programs shown to be effective in RCT into routine care.

The goal of this implementation study was to evaluate MITI as it transitioned from an RCT to real-world settings. Guided by Proctor et al’s framework [29] for implementation research, the study assessed key outcomes in 3 domains—implementation (process), service (clinical and program), and client (satisfaction)—to ensure that MITI as usual care continued to be beneficial and acceptable to an expanded underserved population. The study also conducted qualitative interviews with patients and staff during the transition to learn implementation barriers and facilitators, with the goals of making changes in real time to the MITI program based on the feedback that we learned, and of guiding future implementation efforts. This report summarizes the results of the quantitative implementation evaluation. The results of the qualitative interviews learning of barriers to and facilitators of the implementation process are reported separately.

## Methods

### Study Design

A mixed-methods study was conducted to evaluate the implementation of MITI into routine care at 2 safety-net health care systems in NYC. The study’s quantitative evaluation used a single-group, prepost study design to assess the clinical outcomes, core process outcomes, and patient satisfaction with MITI as it became routine care [29]. Our research team conducted in-depth qualitative interviews with patients and staff assessing barriers to and facilitators of the implementation of MITI.

### Setting

Bellevue Hospital was the site of the original MITI pilot RCT. At the completion of the pilot program, the RCT data were presented to Bellevue’s parent company, NYC Health and Hospitals. NYC Health and Hospitals saw the value in the program and gave approval to transition MITI from a research program to a usual care program available to patients with diabetes needing basal insulin titration in the Bellevue Adult Primary Care Center. For MITI to run effectively, there needed to be a full-time program coordinator, and NYC Health and Hospitals provided this. In 2015, the MITI clinical director (NL) sought to expand MITI to a second site. Gouverneur Health was selected because it is part of NYC Health and Hospitals, an affiliate of NYU, and a clinic that serves the underserved. A common clinical champion made a connection between the MITI program director and the leadership team at Gouverneur Health. Once this connection was made, the Bellevue-based MITI program director met with leadership at Gouverneur Health. The MITI director shared information from the pilot RCT with Gouverneur leadership regarding MITI’s efficaciousness, feasibility, adoptability, and patient satisfaction. The Medical Director, Nursing Director, and clinicians at Gouverneur saw the benefit of implementing this efficacious program that had high patient satisfaction and that did not bring a significant additional time burden to staff. Nursing leadership felt that the work of MITI was well within the scope of the general internal medicine clinic nurses at Gouverneur Health.

Thus, between April 2016 and April 2017, MITI was implemented into usual care in the adult medical clinics of Bellevue Hospital and Gouverneur Health in NYC. As stated above, both facilities are members of the NYC Health and Hospital Enterprise (the largest public hospital system in the United States) and affiliates of the New York University School of Medicine. Bellevue Hospital and Gouverneur Health each care for approximately 5000 patients with diabetes. Approximately 30% of patients with diabetes at Bellevue and 10% of patients with diabetes at Gouverneur have an HbA_1c_ of 8% (64 mmol/mol) or above. Both Bellevue and Gouverneur are safety-net centers serving a multiethnic and multiracial patient population. Most patients (65% at Bellevue, 75% at Gouverneur Health) have either Medicaid or are uninsured.

### Inclusion and Exclusion Criteria

Patients appropriate for MITI have the following: (1) type 2 diabetes; (2) an HbA_1c_ ≥8% or 64 mmol/mol, (based on the labs obtained at enrollment or the most recent value in the electronic medical record within the 2 months before enrollment); (3) FBG levels in the last 2 weeks ≥130 mg/dL (7.2 mmol/L) but ≤400 mg/dL (22.2 mmol/L); (4) to be starting basal insulin or in need of titration of an existing dose of basal insulin; (5) their basal insulin be either glargine or detemir, dosed once daily; (6) a creatinine ≤1.3 mg/dL (115 µmol/L) for women or ≤1.4 mg/dL (123.8 µmol/L) for men; (7) an age between 18 and 70 years (during this study’s evaluation period, an exception was made for 1 patient above the age of 70 years. The patient was inadvertently enrolled by one of the team nurses and it was not brought to the MITI team’s attention until mid-way through the program.); (8) a cell phone; and (9) the ability to receive text messages, to understand the text messages in either English or Spanish, to check their home FBG, to text back the results, and to accept and make phone calls.

Patients were excluded if they did not meet the above inclusion criteria, if they were taking oral steroids at the time of enrollment, if they had taken oral/injectable steroids within the past 2 weeks, or if they were currently taking rapid-acting insulin.

Any patients referred to MITI who had not injected insulin previously were sent for education on how to do so before enrollment.

### Mobile Insulin Titration Intervention Program Referral, Enrollment, and Consent

Providers and nurses at the 2 clinics were educated about the MITI program before implementation. When a patient needed once-daily basal insulin titration, and preferred to receive titration instructions remotely versus coming back to the clinic, providers referred the patient to MITI. Patients were almost always enrolled immediately following an in-person visit. However, there were a few times when the MITI program was notified of a referral for a patient that was not currently in clinic, and program staff arranged for the patient to return for enrollment.

At enrollment, a MITI team member met with the patient to confirm eligibility, administer a survey capturing demographics, explain the program in more detail, and enroll the patient into a secure Web-based messaging platform developed and maintained by Wellpass (Wellpass, Inc, New York, NY) [30]. Messages sent as short message service (SMS) texts from the Wellpass platform are not encrypted. Therefore, patients at enrollment also signed a consent form that gave their written authorization to exchange protected health information (eg, FBG levels) via text.

### Intervention: Text Messages, Monitoring, Titration, and Goals

Every weekday at a patient-specified time, MITI participants received a text message asking, “What was your fasting blood sugar this morning?” Responses were monitored daily for any “alarm” values, defined as FBG <80 mg/dL [, or 4.4 mmol/L, or FBG >400 mg/dL, or 22.2 mmol/L, which were addressed by the monitoring nurses in real time. Registered nurses called all patients once weekly to advise on dose titration using the structured algorithm that the MITI team developed before and tested during the 2013 pilot study [26].

The MITI coordinator also monitored for the presence of daily responses. If patients did not respond with their FBG value 3 days in a row, the MITI program coordinator reached out to the patient via phone calls to understand the reason for the lack of the text message response and problem solve, if needed.

The goal of the program is to find the patient’s OID. OID is defined as the once-daily basal insulin dose that leads to one FBG of 80-130 mg/dL (4.4-7.2 mmol/L) inclusive or that reaches the 50 unit maximum dose. The program ends the week that the patient reaches their OID, when 12 weeks elapse, or when the program terminates early (ie, when a patient actively withdraws from the program, becomes lost to follow-up, or when patient eligibility changes, as occurred with one patient whose doctor decided soon after referral to MITI that rapid-acting insulin should be started). Patients were considered lost to follow-up when the MITI nurse was not able to reach them despite efforts over 3 consecutive weeks.

### Two Different Staffing Models

The 2 sites used different staffing models to serve the following 2 functions: (1) enrolling, and (2) monitoring and titrating. At Bellevue Hospital, the enrollment onto the secure Web platform and into the MITI program was carried out by the on-site MITI program coordinator. The daily monitoring for alarm values and weekly phone calls at Bellevue Hospital were primarily carried out by 1 of the clinic’s 2 diabetes nurses (both are certified diabetes educators and registered nurses). The diabetic nurses had structured time set aside each week to carry out the work of the MITI program.

At Gouverneur Health, there is no full-time onsite MITI coordinator or diabetes nurse. The medicine clinic nurses enrolled patients into MITI as part of the routine clinic discharge process. At Gouverneur Health, these same medicine nurses were also the team members responsible for checking alarm values daily and making titration calls weekly. This monitoring and titration work was carried out as part of the daily workflow.

### Mobile Insulin Titration Intervention Implementation Process

Evidence-based strategies to implement MITI were used at each site [31]. For example, before the rollout of MITI as usual care, a multidisciplinary advisory board was formed at each site to guide implementation. Potential referring providers and MITI nurses were trained about eligibility criteria, enrollment procedures, daily monitoring, and weekly titration calls. MITI nurses attended multiple in-person trainings on how to use the intervention software (eg, logging on to the system, enrolling patients, monitoring daily text messages). The MITI team attended routine staff meetings after the rollout to give updates and get feedback. As new providers and nurses joined the clinics, they were educated individually. The MITI coordinator was available on an ongoing basis to troubleshoot any issues that arose, review standard procedures, answer questions, and provide information on any updates and/or changes to the program.

### Measures and Outcomes

The study measured multiple program outcomes, clinical outcomes, process outcomes, and satisfaction at both sites informed by the Proctor et al model of outcomes in implementation research [29] to study implementation processes and study outcomes in 2 different clinical settings.

#### Mobile Insulin Titration Intervention Program Outcomes

We measured the percentage of patients who achieved their OID and the number of days required to reach OID. We evaluated the frequency of each component of OID (reaching a FBG of 80-130 mg/dL [4.4-7.2 mmol/L] inclusive or reaching the maximum dose of 50 units). We evaluated the percentage of patients that did not reach OID by 12 weeks or for whom the program was terminated early (ie, no longer met eligibility criteria, withdrew from the program, or were lost to follow-up).

#### Mobile Insulin Titration Intervention Cost Analysis

An analysis of potential cost savings was conducted. The cost of the time of nursing, administration, and the MITI program director, calculated from national wage data obtained from the Bureau of Labor Statistics [32], in addition to technology costs (both technology setup fees and individual staff licenses to access the Web platform [30]), were calculated. Savings associated with patient time (also calculated from national wage tables from the Bureau of Labor Statistics [32]) and with the averted in-person medical clinic visit (based on Healthcare Bluebook fee scales [33]) were evaluated.

#### Mobile Insulin Titration Intervention Clinical Outcomes

Additional clinical measures included FBG values on the first day of MITI as well as the day that qualified the patient for completion, rates of hypoglycemia, and, when available, follow-up HbA_1c_ values were abstracted from the patient’s medical record.

Pre-MITI HbA_1c_ blood test values were accepted when obtained within 2 months of enrollment, and post-MITI HbA_1c_ values were accepted between 2 and 6 months after completion of MITI. Ideally, the MITI HbA_1c_ values would be obtained on the day of enrollment and then once again 2-3 months after program termination. However, in real-world settings (and not RCTs), it is the referring clinician who decides if new labs are needed on the day of enrollment or if the HbA_1c_ from the recent past still represents the glycemic control range for the patient in question. Likewise, for the post-MITI HbA_1c_ s, it was not within the scope of the program to bring patients back in for a special blood test that best fit our time frame. Referring providers were in charge of ordering the post-MITI HbA_1c_ blood tests and, although ideally we would have liked to have seen them between 2-3 months after program completion, we accepted values up to 6 months if this was the time frame within which patients were able to return.

#### Mobile Insulin Titration Intervention Process Outcomes

MITI process outcomes were assessed using data collected by the MITI program as part of routine operations. The Wellpass system captures all texts sent and received, which we used to calculate the percentage of texts prompts that received a patient response. We used clinic administrative data to calculate MITI uptake by providers, defined as the percent of providers who referred at least one patient to MITI, as well as to calculate the percentage of weeks that the nurses were able to connect with the patients to provide titration instructions. MITI nurses documented the time it took per patient per week to carry out the titration intervention.

#### Patient Satisfaction and Patient Time Saved

Patients completed a short survey at enrollment, which asked them how long it took them to travel to the clinic and how long they waited for their appointment, which we used to calculate patient time saved when an in-person visit was averted. The MITI coordinator at Bellevue called all MITI patients following completion of the program to assess patient satisfaction with the program. Patient satisfaction surveys evaluated 6 factors using a Likert-type scale: comfort level sharing information through text, preference for clinic, thoughts on the number of text messages (eg, too few, too many, just right), text helpfulness as a reminder to check sugar levels, text helpfulness as a reminder to take insulin, and text helpfulness as a reminder to make healthy food choices. Patients were not compensated for their participation.

### Statistical Analysis

Descriptive statistics were used to summarize sample demographics and quantitative outcomes, stratified by site. Paired *t* tests were used to examine whether there were significant within-group changes in HbA_1c_ and FBG for the MITI group between baseline and at the end of MITI program. A two-sided *P*<.05 was considered to be statistically significant, and all analyses were performed in SAS 9.4 (SAS Institute Inc, Cary, NC). For the cost analysis, the total costs for each patient were subtracted from the total savings for each patient. These net savings are presented in terms of “per-patient per-week.” To understand net savings on a larger scale, we took these “per-patient per-week” savings and calculated how they would change based on various projections of how many patients would utilize the program in 1 year.

## Results

### Patient Characteristics

**Figure 1:**
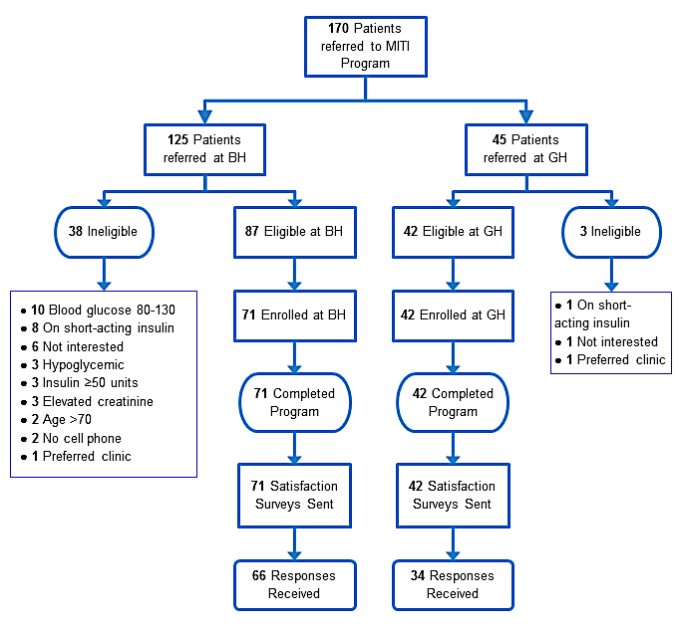
Mobile insulin titration intervention (MITI) program referrals and enrollment diagram. BH: Bellevue Hospital; GH: Gouverneur Health.

Figure 1 displays patient flow through MITI. Across the 2 sites, providers referred 170 patients to the MITI program—129 of whom (75.9%) were eligible. Of these, 113 (87.6%) enrolled. A total of 41 patients were excluded for the following reasons: 10 had a FBG between 80-130 mg/dL (4.4-7.2 mmol/L) in the past 2 weeks, 9 were on short-acting insulin, 3 had any blood glucose (fasting or non-fasting) <80 mg/dL (4.4 mmol/L) in the past 2 weeks, 3 were already taking ≥50 units of insulin, 3 had elevated creatinine, 2 preferred clinics, 2 were older than 70 years, 2 did not have a cell phone, and 7 declined to participate for other reasons. Of the remaining 16 patient referrals, 15 were lost to follow-up and 1 was enrolled in MITI’s year 2 (Figure 1).

Table 1 shows MITI patient demographics. MITI patients had an average age of 50 years (standard deviation, SD=10), 45.1% were female, 78.8% Hispanic, 42.5% unemployed, and 46.0% uninsured. Moreover, 59% chose Spanish as their text language and 40.7% chose English. The initial FBG value was 209 mg/dL (11.6 mmol/L, SD 71) and the mean initial HbA_1c_ level was 11.4% (101 mmol/mol, SD 1.9%). Patients spent on average 142 min (SD 69) traveling to and from the clinic and waiting in the waiting room (52 min travel time each way, SD 27, 43 min waiting room time, SD 39).

### Mobile Insulin Titration Intervention Cost Analysis

A cost analysis indicated that the value of patients’ time and the cost of the averted clinic visits outweighed the cost of the MITI program. The per-patient per-week savings at Bellevue Hospital ranged from US $169.65, if 71 patients per year (the actual number of patients that participated in MITI during this study) participated in the program, to US $176.91 if 100 patients per year participated in the program, to US $185.80 if 200 patients per year participated in the program. At Gouverneur Health, the per-patient per-week savings ranged from US $0.94, if 42 patients participated in the program per year (the actual number of patients that participated in MITI during this study), to US $107.55 if 100 patients participated in the program per year, to US $146.15 if 200 patients participated in the program per year.

### Mobile Insulin Titration Intervention Program Outcomes

Table 2 shows MITI program outcomes related to the frequency of each program discharge status (eg, optimal insulin dose, 12 weeks without optimal insulin dose, program terminated early). In total, 84.1% of patients reached their optimal insulin dose, and they did so in an average of 24 days. This included 74.3% of patients who reached a once-daily basal insulin dose to bring one blood glucose down to between 80 and 130 mg/dL (4.4-7.2 mmol/L), inclusive, as well as an additional 9.7% of patients who reached the maximum dose of 50 units. Moreover, 8.0% of patients did not reach the goal by 12 weeks and 8.0% of patients terminated the program early (1 patient had short-acting insulin added to their regimen and thus became ineligible, 1 decided she did not want to take insulin, 1 patient actively withdrew, and 6 were lost to follow-up and could not be reached despite multiple attempts over 3 weeks).

**Table 1:** Enrolled Mobile Insulin Titration Intervention (MITI) patient demographics.

Demographics		Bellevue (N=71)	Gouverneur (N=42)	Total (N=113)
**Age**				
	Mean age (SD; min-max)	50 (10; 26-69)	50 (11; 24-73)	50 (10; 24-73)
Female gender, n (%)		34 (48)	17 (41)	51 (45.1)
**Race, n (%)**				
	Native Hawaiian or Pacific Islander	0 (0.0)	0 (0.0)	0 (0.0)
	Native American or Alaskan Native	1 (1)	1 (2)	2 (1.8)
	Asian	5 (7.0)	3 (7)	8 (7.1)
	White	7 (10)	3 (7)	10 (8.8)
	Black or African American	8 (11.3)	3 (7)	11 (9.7)
	Other^a^	50 (70)	32 (76)	82 (72.6)
Hispanic ethnicity^a^, n (%)		54 (76)	35 (83)	89 (78.8)
No employment, n (%)		36 (51)	12 (29)	48 (42.5)
No health insurance, n (%)		39 (55)	13 (31)	52 (46.0)
Had a visit co-payment? Yes, n (%)		34 (489)	14 (33)	48 (42.5)
**Language used for text messages, n (%)**				
	Spanish	41 (58)	26 (62)	67 (59.3)
Pre-MITI patient travel and wait time in min, mean (SD)		157 (66)	117 (66)	142 (69)

^a^All patients who checked the “Other” race option said they were Hispanic.

**Table 2:** Mobile Insulin Titration Intervention (MITI) program outcomes.

MITI discharge status			Bellevue (N=71)			Gouverneur (N=42)			Total (N=113)		
			n (%)	Mean days in MITI (min-max)	SD	n (%)	Mean days in MITI (min-max)	SD	n (%)	Mean days in MITI (min-max)	SD
**Achieved optimal insulin dose**			57 (80)	26 (3-56)	24	38 (91)	21 (3-77)	22	95 (84.1)	24 (2-84)	23
		Reached 80-130	50 (70)	21 (3-84)	20	34 (81)	17 (2-42)	17	84 (74.3)	20 (2-84)	19
		Reached max insulin dose (50 units)	7 (10)	62 (27-84)	22	4 (10)	57 (10-77)	32	11 (9.7)	60 (10-84)	25
Not at goal by 12 weeks			7 (10)	84	N/A^a^	2 (5)	84	N/A	9 (8.0)	84	N/A
Program terminated early			7 (10)	35 (16-63)	18	2 (5)	37 (16-58)	30	9 (8.0)	36 (16-63)	19

^a^N/A: not applicable.

**Table 3:** Mobile insulin titration intervention (MITI) clinical outcomes for fasting blood glucose values.

MITI clinical outcomes		Bellevue (N=70^a^)	Gouverneur (N=42)	Total (N=112^a^)
**Mean fasting blood glucose value**				
	First day of MITI in mg/dL (SD), mmol/L (SD)	214 (73), 11.9 (4.2)	201 (68), 11.2 (4.1)	209 (71), 11.6 (4.2)
	Last day of MITI in mg/dL (SD), mmol/L (SD)	144 (48), 8.0 (2.9)	136 (41), 7.6 (2.8)	141 (45), 7.8 (3.0)
*P* value		<.001	<.001	<.001

^a^There are missing data for one Bellevue patient who never texted.

**Table 4:** Mobile insulin titration intervention (MITI) clinical outcomes for HbA_1c_ values.

MITI clinical outcomes	Bellevue (N=51^a^)	Gouverneur (N=29^a^)	Total (N=80^a^)
Mean pre-MITI A_1c_ in % (SD), mmol/mol (SD)	11.6 (1.8), 103 (20)	11.1 (2.0), 98 (22)	11.4 (1.9), 101 (21)
Mean post-MITI A_1c_ in % (SD), mmol/mol (SD)	10.3 (2.1), 89 (23)	9.4 (2.2), 79 (24)	10.0 (2.2), 86 (24)
*P* value	<.001	<.003	<.001

^a^Data are only included for those patients who have both a pre- and post-HbA_1c_.

### Mobile Insulin Titration Intervention Clinical Outcomes

Table 3 shows MITI clinical outcomes related to changes in fasting blood glucose values. The mean FBG on the first day of the program was 209 mg/dL (11.6 mmol/L, SD 71) and it fell to 141 mg/dL (7.8 mmol/L, SD 45) on the day that qualified the patient for completion of the program, *P*<.001. Of note, out of 2049 texted blood glucose values (and any additional information shared during the weekly phone calls with the MITI nurses), there were only 2 reports of hypoglycemia; neither were severe.

Moreover, 80 of the 113 MITI patients have had HbA_1c_ values within both windows for pre and post lab collection and were included in our main HbA_1c_ analysis (Table 4).

The breakdown of when the 80 pre HbA_1c_ lab values were collected is as follows: 20 on the day of enrollment, an additional 22 were collected within 1 week before enrollment, another 11 were collected within 2 weeks of enrollment, and the remaining 27 within 2 months of enrollment. The average time of pre HbA_1c_ lab draw was within 17.2 days of enrollment. Post HbA_1c_ lab collection had to be within 6 months of program completion date. The review found that 34 of the 80 patients had their post HbA_1c_ labs collected within 3 months of program completion. The remaining 46 had labs collected by the end of the 6-month window. The average time of post HbA_1c_ lab collection was at 104.1 days after program completion.

HbA_1c_ levels fell from a mean of 11.4% (101 mmol/mol, SD 1.9%) at enrollment (SD 21) to 10.0% (86 mmol/mol, SD 2.2%) at follow-up (SD 24), *P*=.003. Moreover, 43 of the 80 patients had an HbA_1c_ that fell by at least 1% point. In addition, 29 of the 80 patients had an HbA_1c_ that fell by ≥2% points. Furthermore, 17 the 80 patients with HbA_1c_ results have an HbA_1c_ that fell to ≤8% (64 mmol/mol).

Of note, the pre HbA_1c_ mean for the 96 of 113 enrollees who had a baseline HbA_1c_ within 2 months of enrollment (not just of the 80 that had *both* pre and post values) had essentially the same mean of 11.6% (103 mmol/mol), supporting that those that have had follow-up data available in the electronic medical record were representative of the entire population in terms of disease severity at the start of MITI.

### Mobile Insulin Titration Intervention Process Outcomes

Table 5 shows MITI process outcomes. We found that 90.1% of MITI’s text message prompts received a response, demonstrating a very high acceptability and participation rate for those enrolled in the MITI program. Review of the data showed that nurses were able to connect with patients to provide titration instructions 81.9% of the time. Median nurse time was 15 min per patient per week to carry out the titration intervention, both at Bellevue Hospital and Gouverneur Health. This includes the time required to prepare for the weekly titration phone call, make the phone call (which often included an interpreter being on the line), and to document the phone call.

When examining the proportion of providers who made at least one referral to MITI, we found that 83% of attending physicians, 42% of resident physicians, 62% of physician assistants, and 100% of both diabetes educators at Bellevue made at least one referral (Table 6)

**Patient Satisfaction Outcomes**

Table 7 shows patient satisfaction data. Patient satisfaction surveys revealed that 97.0% of patients were comfortable sharing their information through text, 97.0% preferred not having to come to clinic, and 94.0% thought that the number of texts received were just right (of note, the other 6% thought they were too few). Surveys also showed that 96.0% felt that the texts were somewhat or very helpful as reminders to check their blood glucose, 84.0% felt the texts were somewhat or very helpful as reminders to take their insulin, and 87.0% felt the texts were somewhat or very helpful as reminders to make healthy food choices.

**Table 5:** Mobile insulin titration intervention (MITI) process outcomes.

MITI process outcomes	Bellevue	Gouverneur	Total
Text response rate (responses ÷ prompts), n (%)	1387/1530 (90.65)	662/744 (89.0)	2049/2274 (90.11)
Call connection rate (connections ÷ weeks), n (%)	264/340 (77.6)	152/168 (90.5)	416/508 (81.9)
Median nurse time (min) for weekly titration interaction^a^ (per-patient per-week), mean (SD)	15 (7)	15 (6)	15 (7)

^a^Titration Interaction time includes the time to prepare for the call, have the call (often with a translator), and document the call.

**Table 6:** Percentage of providers making at least one referral to the Mobile Insulin Titration Intervention (MITI) program.

Provider type	Bellevue, n (%)^a^	Gouverneur, n (%)^a^	Total, n (%)^a^
Attending physician	28 (97)	13 (68)	41 (85)
Resident	37 (40)	4 (15)	39^b^ (42)
Physician assistant	3 (50)	5 (71)	8 (62)
Diabetes nurses	2 (100)	N/A^c^	2 (100)

^a^Referral rates calculated as the number of unique referring clinicians divided by the total number of possible referring clinicians.

^b^A small number of residents rotated through and made referrals at both sites during the study period.

^c^N/A: not applicable.

**Table 7:** Mobile insulin titration intervention (MITI) patient satisfaction and program feedback among patients who have completed the program.

Patient experience questions		Bellevue (N=66 survey respondents)	Gouverneur (N=34 survey respondents)	Total (N=100 survey respondents)
**Comfort level sharing info via texts, n (%)**				
	Very	65 (99)	32 (94)	97 (97.0)
	Somewhat	0 (0)	2 (6)	2 (2.0)
	Not at all	1 (2)	0 (0)	1 (1.0)
**Preference for clinic, n (%)**				
	No	65 (99)	32 (94.)	97 (97.0)
	Yes	1 (2)	2 (6)	3 (3.0)
**Number of texts were, n (%)**				
	Too many	0 (0)	0 (0)	0 (0.0)
	Just right	64 (97)	30 (88)	94 (94.0)
	Too few	2 (3)	4 (12)	6 (6.0)
**Texts helpfulness as reminder to check sugar levels, n (%)**				
	Very	48 (72)	18 (53)	66 (66.0)
	Somewhat	16 (24)	14 (41)	30 (30.0)
	Not at all	2 (3)	2 (6)	4 (4.0)
**Texts helpfulness as reminder to take insulin^a^, n (%)**				
	Very	24 (36)	10 (29)	34 (34.0)
	Somewhat	33 (50)	17 (50)	50 (50.0)
	Not at all	9 (14)	7 (21)	16 (16.0)
**Texts helpfulness as reminder to make healthy food choices, n (%)**				
	Yes, often	39 (59)	16 (47)	55 (55.0)
	Sometimes	20 (30)	12 (35)	32 (32.0)
	Never	7 (11)	6 (18)	13 (13.0)

^a^Patients who said that the texts were not helpful as a reminder to take insulin said that they were already accustomed to taking their insulin each day before the program.

### Technology

Occasionally during the course of the program, there was a random day where the text messages were not delivered. The MITI program coordinator (who checks daily to make sure the program is running) noted this when it occurred and reached out to Wellpass to troubleshoot the issues. It was always remedied quickly.

## Discussion

### Principal Findings

This study evaluated the implementation of a mobile intervention to titrate basal insulin for uncontrolled type 2 diabetes patients (“MITI”), as it became usual care at 2 ambulatory clinics in NYC. The study produced several important findings that help address existing literature gaps regarding the real-world implementation of telemedicine interventions, particularly in resource-poor settings such as US safety-net clinics [27]. A strength of the study design was its evaluation of MITI as it transitioned into usual care at 2 sites that had different patient populations and very different staffing models. Bellevue Hospital used a designated program coordinator to enroll patients into the MITI program and then specialty trained diabetes nurses to carry out MITI’s clinical work (daily monitoring and weekly titration phone calls). Gouverneur Health used the general medical clinic registered nurses to both enroll patients (as part of the routine clinic outtake process) and to carry out MITI’s clinical work. Both sites found success, showing MITI to be adaptable and flexible in different settings. The latter setting, in particular, highlighted MITI as generalizable to general medical clinics where registered nurses (but perhaps not program coordinators nor specialty trained diabetes nurses) will be present.

Program and clinical data (ie, percent of patients reaching OID, time to OID, changes in FBG and HbA_1c_ levels) showed that patients enrolled in the MITI program achieved excellent clinical outcomes that are very similar to those found in MITI’s prior pilot RCT [26]. This demonstrates that MITI was able to achieve continued positive outcomes in real-world settings using regular clinical staff serving expanded safety net populations. Moreover, the study of a patient population receiving MITI as usual care shows that MITI is able to produce positive outcomes with patients who did not self-select to participate in a controlled research study.

Process outcomes (ie, text and titration call response rates) further demonstrated high patient engagement, acceptability, and usability of the program among patients. Previous studies have reported that diabetes patients find telehealth interventions easy to use and that they engage with text-messaging interventions, and prior implementation research has shown that employing user-friendly technology that takes little time to learn and use in telemedicine interventions for diabetes patients is a key component of implementation success [34]. In this study, patients were easily able to use MITI’s once-daily basic SMS messaging and brief weekly phone calls. Additionally, satisfaction survey responses found that most patients were satisfied with the number of texts received, were comfortable sharing their information through text, and preferred not having to come to the clinic. Although the MITI daily text only included the question, “What was your fasting blood sugar this morning?”, without additional motivational or educational content, patients repeatedly shared on surveys that just knowing that the text message was coming in the morning provided the motivation to make healthier food choices, take their insulin, and check their blood glucose in the morning. Patient satisfaction ratings with MITI were similar to—or greater than—prior research evaluating telemedicine interventions for diabetes patients [35-37]. For example, Odnoletkova et al found that 98% of patients enrolled in a telecoaching intervention for diabetes patients reported overall satisfaction with the program and 92% felt that phone was an acceptable mode of communication [35]. Welch and Balder found high acceptability, convenience, and ease of use ratings (>80%) from 30 diabetes patients enrolled in a multi-component telehealth program being studied at an urban community center. Of note, prior reported data on the acceptability of telemedicine interventions for diabetes patients have largely been collected during feasibility or controlled studies [37]. This implementation study makes a significant contribution to the literature on telemedicine interventions for diabetes patients by demonstrating high patient acceptability and usability of MITI in a real-world, safety-net setting.

Low adoption by providers can be one of the greatest barriers to implementation of telehealth interventions, yet our referral data showed high adoption of MITI by staff, with most providers making at least one MITI referral. This report did not assess acceptability among providers. Detailed information about acceptability will be reported in a separate manuscript. Briefly, interviewed providers who made at least one referral reported that their perception of MITI as an effective, convenient, usable, and acceptable option for their patients drove their adoption of the intervention (consistent with other implementation studies evaluating factors that influence provider use of new telehealth programs) [35,38,39]. Interviewed providers who did not refer a single patient provided reasons such as patients neither English- nor Spanish-speaking (the only two languages our program was able to offer) or older and less technologically “savvy.” Prior implementation research has also suggested that providers may not refer patients to mHealth interventions because of their concerns about the effectiveness of mobile health care as compared with direct patient contact [34].

Finally, process outcomes showed the program to be feasible as observed nurse titration time of 15 min per patient per week was reasonable. The study’s cost-savings analysis further showed that the MITI program’s savings in terms of visits averted and patient time saved far outweighed the costs of technology and staff time at both sites. Of note, the per-patient per-week savings are less at Gouverneur Health because the technology company that we worked with charges a license for each clinician that accesses the platform. At Bellevue Hospital, there were only 5 team members that needed to access the platform. These 5 team members included the 2 diabetes nurses who ran the program for all MITI patients, 1 general medical clinic nurse (who was a backup in case both diabetes nurses were away), and the MITI clinical coordinator and program director (both of whom oversaw the program at both locations, but spent the majority of their combined time at Bellevue Hospital). In contrast, at Gouverneur Health, there were 18 nurses that participated in the MITI program, and each of them needed their own license. Of note, this cost sensitivity analysis is based on the pricing of one Web platform provider. In addition, this cost analysis does not take into account potential downstream health savings from improved glycemic control.

### Limitations

Similar to many implementation research studies, our study had no control group for the clinical outcomes. However, the original MITI pilot RCT showed that MITI was efficacious compared with usual care [26]. Nurse time spent on the project was based on self-report, which may be subject to recall bias. Moreover, we only had pre and post HbA_1c_ data on 71% of MITI patients. It is possible that there was a clinical difference between these patients, and the 29% of patients who did not have pre and post labs within the lab window. Additionally, a further limitation of this study might be our gap of knowledge in the potential reach of MITI. It is hard for us to know how many patients would have been eligible for the program, as there is no registry of such patients.

### Conclusions

MITI is a patient-centered, text message-based program that allows the remote titration of once-daily basal insulin solely through the use of basic cell phone technology, which has great potential to improve access to care and reduce disparities in diabetes care for a multiracial, multiethnic, low-income population. This implementation study showed MITI to have continued success after transitioning from an RCT pilot program into real-world settings. MITI was found to flexible in different settings, generalizable to a general medical clinic setting, highly acceptable to patients and providers, and feasible for nurses to deliver as part of their routine workflow.

## Abbreviations

BH: Bellevue Hospital
FBG: fasting blood glucose
GH: Gouverneur Health
HbA_1c_: glycated hemoglobin
MITI: mobile insulin titration intervention
OID: optimal insulin dose
SMS: short message service
